# Use of wearable technology for performance assessment: A validation study

**DOI:** 10.1016/j.medengphy.2015.03.017

**Published:** 2015-07

**Authors:** Enrica Papi, Denise Osei-Kuffour, Yen-Ming A Chen, Alison H McGregor

**Affiliations:** a Department of Surgery and Cancer, Imperial College London, London, UK; b Centre for Bio-Inspired Technology, Institute of Biomedical Engineering, Imperial College London, London, UK

**Keywords:** Wearable sensor, Osteoarthritis, Rehabilitation, Acceleration, Exercise, Performance, OA, osteoarthritis, FTSST, five time sit-to-stand test, AWS, attachable wearable system, IMU, inertial measurement unit, RPSIS, right posterior superior iliac spine, RGT, right greater trochanter, FFT, Fast Fourier transform

## Abstract

The prevalence of osteoarthritis is increasing globally but current compliance with rehabilitation remains poor. This study explores whether wearable sensors can be used to provide objective measures of performance with a view to using them as motivators to aid compliance to osteoarthritis rehabilitation. More specifically, the use of a novel attachable wearable sensor integrated into clothing and inertial measurement units located in two different positions, at the waist and thigh pocket, was investigated. Fourteen healthy volunteers were asked to complete exercises adapted from a knee osteoarthritis rehabilitation programme whilst wearing the three sensors including five times sit-to-stand test, treadmill walking at slow, preferred and fast speeds. The performances of the three sensors were validated against a motion capture system and an instrumented treadmill. The systems showed a high correlation (*r*^2^ > 0.7) and agreement (mean difference range: −0.02–0.03 m, 0.005–0.68 s) with gold standards. The novel attachable wearable sensor was able to monitor exercise tasks as well as the inertial measurement units (ICC > 0.95). Results also suggested that a functional placement (e.g., situated in a pocket) is a valid position for performance monitoring. This study shows the potential use of wearable technologies for assessing subject performance during exercise and suggests functional solutions to enhance acceptance.

## Highlights

• We assessed three sensors in monitoring activity performance.
• A novel flexible sensor system was validated.
• A functional sensor placement is as valid as a more conventional one.
• A frequency domain approach was successfully applied to evaluate outcome measures.

## 1 Introduction

Osteoarthritis (OA) represents one of the most common forms of musculoskeletal disorders affecting predominately load bearing joints [1]. It ranks as the second leading cause of disability and the fastest growing major health condition [2]. Estimates show that more than 250 million people worldwide are affected by OA of the knee [3] and this number is expected to increase in relation to expanding ageing population, increased obesity and lack of physical activity [2,4].

OA is currently managed through a combination of lifestyle modifications, pain-killing treatments and rehabilitation exercises aimed to improve function. Ultimately surgical joint replacement can be performed but this is normally reserved for cases of advanced joint degeneration. Implants are however, costly, invasive and have a limited life-span. As people live longer the likelihood that their joint replacement will last for the duration of their lifetime reduces. Thus there is a need to provide more effective earlier intervention to delay the need for joint replacements. Clinical guidelines recommend the use of regular exercise to enhance joint function, alleviate pain and delay the need for surgical intervention [5]. However, research has shown that both compliance with and attendance at rehabilitation classes is poor [6,7] compromising the effectiveness of the treatment. Reasons for poor treatment fidelity include a lack of understanding of the content, organisational issues such as location and time causing conflict with everyday commitments, but also the individuals’ inability to perceive change in function hampered further by the limited availability of markers of improvement or progression [7,8]. Markers of improvement are important tools to motivate patients whilst exercising and also represent important outcome measures for clinicians. The use of portable technology to support rehabilitation is an emerging concept that could increase the availability and accessibility to treatments and ultimately their effectiveness. Developments in portable sensing technologies offer the possibility to track and analyse body movements outside of the laboratory environments, potentially permitting their use in rehabilitation.

Currently, inertial sensors are used as an alternative to laboratory-based systems to monitor activities of daily living, assess gait and body segment movements, and to evaluate postural control and balance [9]. Being portable, inertial sensors allow remote monitoring in real-life environments as opposed to the artificial and confined laboratory space. The bulkiness of the system adopted depends upon the complexity of the data to be measured, thereby dictating the choice of sensor or sensors if multiple body segments are being measured.

The use of wearable and portable technologies are being explored in clinically-oriented research studies, but to date have not been deployed in rehabilitation practices. The main reason for this is a mismatch between the technology and the users (patients and clinicians) limiting clinical uptake. A recent systematic review [10] highlighted patient and clinician preferences for body worn sensor devices. It was elicited that for the patient and the clinicians, it is important that the sensors are compact, and embedded if possible, so as to have minimal effect on their daily routines.

The exploratory study reported here investigated the use of portable sensing technology to monitor performance of rehabilitation exercises. The specific aims were:

1. To determine the validity of a novel attachable wearable sensor system, to monitor a subject's performance during exercising.
2. To explore whether a functional positioning of an inertial measurement unit (IMU) compromises the ability of the system to monitor activities compared to a more conventional positioning.

It was hypothesised that the outputs from the portable systems used would correlate to the relative gold standard measurement, validating the use of these sensors for performance monitoring.

## 2 Methods

### 2.1 Participants

Fourteen able-bodied subjects volunteered to participate in the study; including seven males and seven females, with a mean age of 25 (SD 8) years, height of 1.71 (SD 0.09) m and body mass of 68.1 (SD 12.0) kg. The protocol was approved by the College Research Ethics Committee and all subjects gave written informed consent.

### 2.2 Test protocol

Participants were asked to complete a five time sit-to-stand test (FTSST) and to walk on a treadmill whilst wearing three sensors. These tasks were selected from a routine exercising programme for patients with OA knees. For the FTSST, each subject was asked to perform five consecutive sit-to-stand and stand-to-sit cycles with their arms crossed over the chest as fast as they were able to. A chair with a height and depth of 40 cm by 40 cm, without arm rests and no back was used. Each subject repeated the test three times following standardised instructions [11]. The time taken to complete each FTSST was evaluated.

The walking task comprised treadmill walking at a self selected speed determined during 6 m timed over ground walk, and at slow speed (0.5 time the self selected speed). Data were collected for 40 s for each trial. For a sub group of eight participants a fast speed walking was also evaluated. The fastest tolerated walking speed for each subject was considered and defined as the speed at which the participant felt comfortable walking (almost running) without the support of handrails whilst still maintaining a period of double limb support [12]. This was determined experimentally for each subject by gradually increasing the treadmill speed. Before commencing data acquisition each subject was given time to acclimatise to the treadmill (6 min at 4 km/h) [13]. They were encouraged to wear their regular footwear/trainers for the experiment. Stride time and length were evaluated for the walking tasks.

### 2.3 Instrumentation

**Fig. 1:**
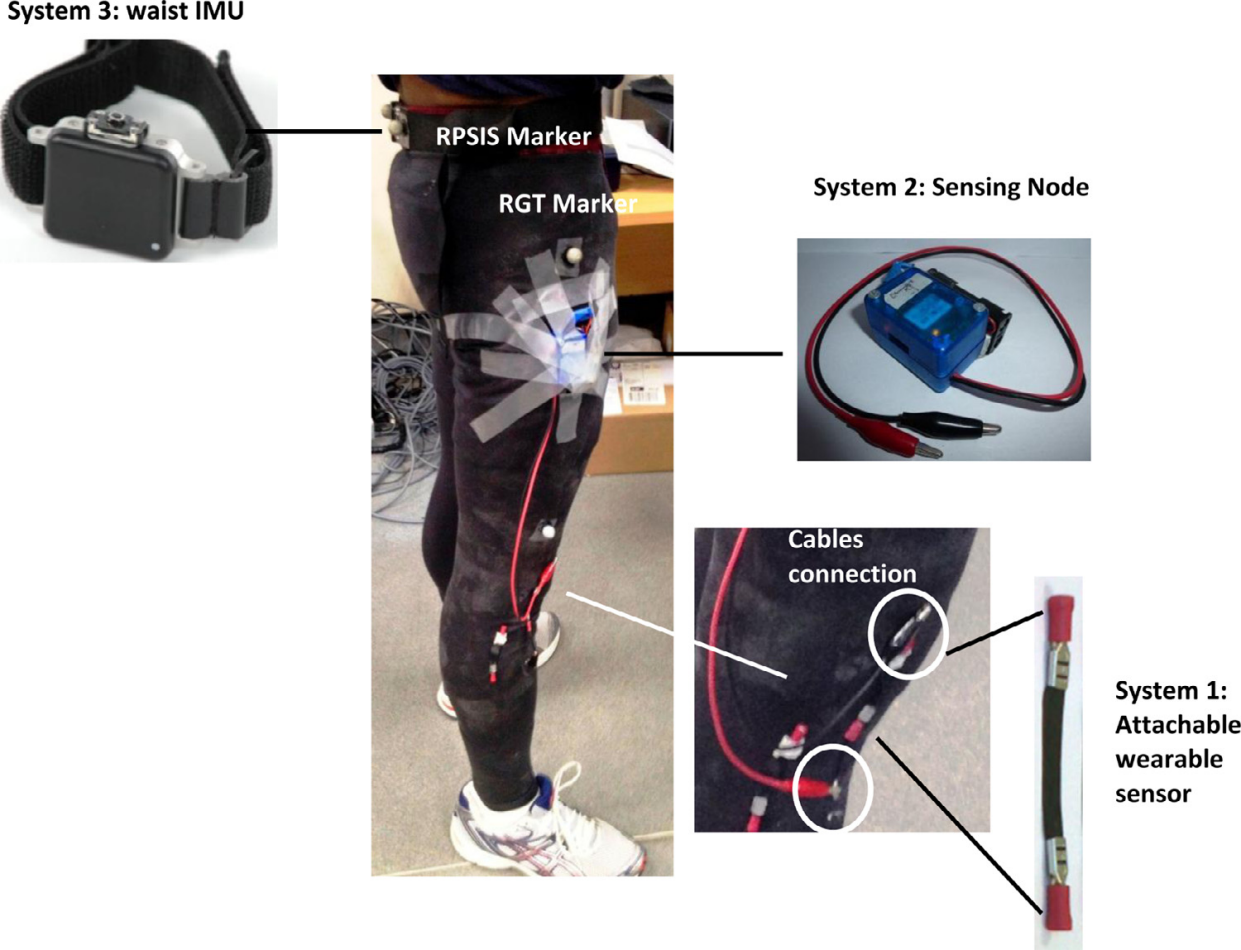
Participant set-up during the test. Systems positioning is visible as well as markers attachment on the right greater trochanter (RGT) and right posterior iliac spine (RPSIS). Although two attachable sensors are present in the photo, only one was connected via cables to sensing node and used for the tests.

Three portable sensor systems were used to objectively assess tasks performances: an attachable wearable system (AWS), and two inertial measurement units (IMUs) (Fig. 1). The AWS (system1) comprises a flexible sensor unit sewed into a tight-fitting trouser garment and positioned over the lateral aspect of the right knee. The positioning was adjusted to fit each subject's underlying knee anatomy. The sensor unit consists of a rectangular piece of composite material (50 mm × 100 mm, thickness < 0.2 mm, mass < 10 g) made from 20% conductive carbon black nanopowder and 80% polyurethane allowing a resistor-like functioning [14]. A change in resistance occurs every time a force is exerted on the material. Based on this principle, the AWS can be used to detect and sense knee motion.

Data output from the AWS was acquired via synchronisation with a custom built wireless sensing node (system 2). Although systems 1 and 2 are synchronised, meaning they share the same Bluetooth module to transmit data, their measurements are separate and do not influence each other in any way. The node consists of three independent printed circuit board tiers. The core tier accommodates the microprocessor (64 MHz PIC18F family, Microchip Technology Inc., Chandler, AZ, USA) and an IMU system with a 3-axis accelerometer (ADXL345, Analog Devices Inc., Norwood, MA, USA) and 3-axis gyroscope (L3G4200D, STMicroelectronics, Geneva, Switzerland). The AWS tier hosts the analogue interface circuitry for the flexible sensor, to which it was physically connected via short cables. The connectivity tier is dedicated to a Bluetooth module (RN42, Microchip Technology Inc., Chandler, AZ, USA) allowing wireless data acquisition at 122 Hz synchronously from the IMU and AWS. Data were transmitted to a laptop (HP EliteBook, Hewlett-Packard Company, Palo Alto, CA, USA) and acquired via a customised C++ interface. The node runs off a 3 V battery and is encased in a box of 3 × 50 × 40 mm (width × length × height) dimensions and with a mass of 54 g approximately. The unit was positioned on the thigh at the level of each subject's right greater trochanter using tape. This position was chosen to simulate the subject's pocket with the intent to replicate an everyday functional placement.

The third sensor was a waist-worn OpalTM (APDM Inc., Portland, OR, USA) IMU (system 3, mass: 22 g, dimensions: 48.4 × 36.5 × 13.4 mm) that also encases a 3-axis accelerometer and 3-axis gyroscope. Data were collected using the logging mode at 128 Hz using APDM software as per manufacturer instructions. This system was positioned at a level between the third and fourth lumbar vertebrae with a clip belt. This positioning is common among studies that use accelerometry [15–20].

A 10 camera optical tracking system (Vicon, Oxford Metrics Ltd., Oxford, UK) was used as the reference system to validate the portable sensors for monitoring the FTSST. Data about the 3D positioning of spherical (14 mm diameter) retro-reflective markers were collected at 100 Hz. The markers, to allow standardisation between subjects, were positioned on the right posterior superior iliac spine (RPSIS) close to system 3, on its waist band, and on right greater trochanter (RGT) close to system 2 (Fig. 1). Markers trajectories were used to determine the start and stop of the FTSST task and hence the reference value for its duration.

An Instrumented treadmill (h/p/Cosmos Gaitway, h/p/cosmos sports & medical gmbh, Nussdorf-Traunstein, Germany) was used as the reference for stride parameters calculation. The treadmill uses data from two built in force plates and its bespoke software calculates gait related parameters.

### 2.4 Data analysis

The accelerometry data, from both IMU systems, were used in the current analysis. Only the anterior/posterior acceleration signal was considered as it was found to be the most revealing and repeatable signal between subjects. Markers trajectories were output using Nexus software (Vicon, Oxford Metrics Ltd., Oxford, UK) and filtered using Woltring's general cross-validatory quintic smoothing spline with a predicted mean square error of 15 mm [21]. Subsequent data analysis was performed using Matlab software (The MathWorks Inc., Natick, MA, USA). Accelerometer data were low-pass filtered (fourth-order recursive Butterworth filter) at a cut-off frequency of 3 Hz [15–17]. The same filtering with a cut-off frequency of 1 Hz was applied to the AWS outputs to remove excessive sensor noise without any loss of signal integrity.

A peak detection algorithm was used to calculate FTSST duration from the anterior/posterior acceleration signals of the waist and thigh IMUs. For the AWS, a frequency domain approach was used to generate the FTSST duration, as no clear patterns were observed among subjects to define a generalised peak detection algorithm. A fast Fourier transform (FFT) algorithm was implemented to identify the frequency content of the AWS output. The FTSST is the periodic repetition of one sit-to-stand and stand-to-sit cycle performed five times. Through the FFT, the fundamental frequency which corresponds to one period (*f* = 1/*T*) can be determined and the FTSST duration defined as five times the period. The reference values of FTSST duration were obtained from the analysis of markers movements. An algorithm, that used thresholds defined from markers vertical displacements and vertical velocities, was used to identify the start and end of the task. The RPSIS marker was used to define the FTSST duration as reference for the waist IMU output and the RGT marker defined the reference value for the thigh IMU and AWS. Different reference values were considered as the thigh IMU and AWS can detect leg movement, which will not be necessarily simultaneous to trunk movement [22].

A frequency domain approach was used to determine stride time and stride length from all three systems. Walking, on the treadmill at constant speed, is the periodic repetition of consecutive strides. The FFT was used to identify the fundamental frequency of one period which defined the stride time. For the waist accelerometer the periodic movement is a step, as the positioning allows a detection of both leg movements, and thus the calculated fundamental frequency from FFT corresponds to the frequency of a step. By definition a stride includes two steps, and assuming gait symmetry for this study, the frequency of a stride is half the step frequency. The stride length was calculated by dividing the speed (Section 2.2) by the frequency of a stride. Stride time and stride length values as defined by FFT approach were compared to the treadmill calculated values.

### 2.5 Statistical analysis

Descriptive statistics (mean (SD)) were used to summarise the results. To validate the ability of the sensors to measure FTSST duration, stride time and stride length, values were compared to the relative reference parameters. Correlation (*r*^2^) between measurements was calculated and the level of agreement between each of the sensors and the gold standard tools was verified using the Bland Altman method [23]. The accuracy of the systems was evaluated in terms of root mean squared errors (RMSEs). Finally, inter sensor reliability was assessed using intra class correlation coefficients [24]. Statistical analysis was computed using Matlab Statistics Toolbox (The MathWorks Inc., Natick, MA, USA).

## 3 Results

**Fig. 2:**
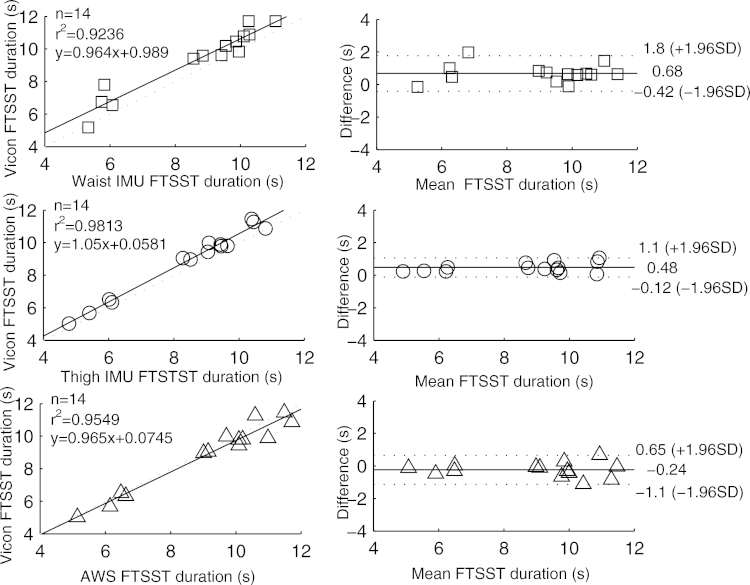
Correlation and Bland Altman plot of agreement for the waist IMU (□), thigh IMU (◯) and AWS (^) against Vicon reference for FTSST duration. Horizontal lines represent the mean difference and the upper and lower limit of agreements (dotted line).

**Fig. 3:**
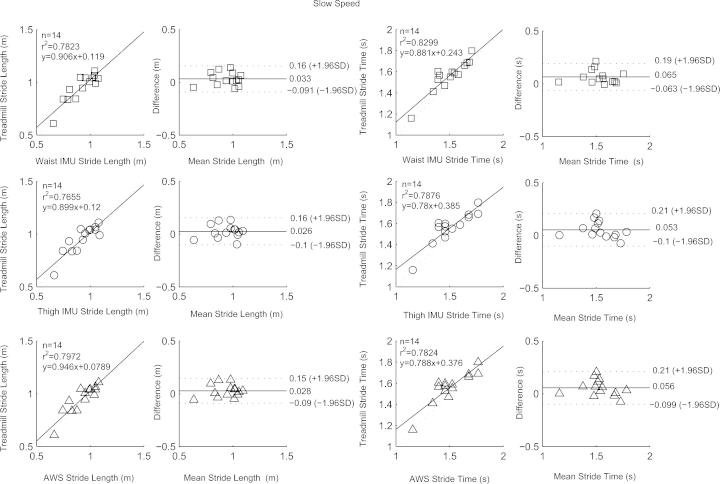
Correlation and Bland Altman plot of agreement for the waist IMU (□), thigh IMU (◯) and AWS (^) against estimated parameters by the treadmill for stride time and length at slow speed. Horizontal lines represent the mean difference and the upper and lower limit of agreements (dotted line).

**Fig. 4:**
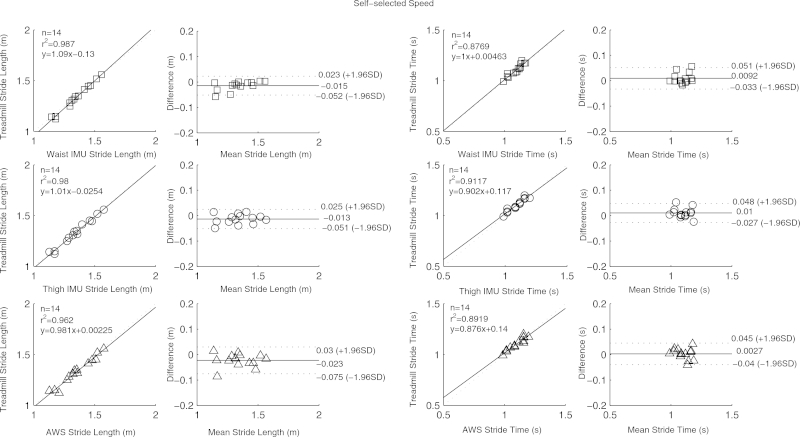
Correlation and Bland Altman plot of agreement for the waist IMU (□), thigh IMU (◯) and AWS (^) against estimated parameters by the treadmill for stride time and length at self-selected speed. Horizontal lines represent the mean difference and the upper and lower limit of agreements (dotted line).

**Fig. 5:**
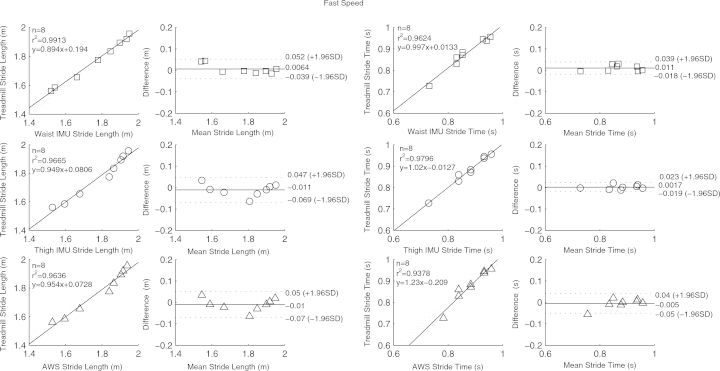
Correlation and Bland Altman plot of agreement for the waist IMU (□), thigh IMU (◯) and AWS (^) against estimated parameters by the treadmill for stride time and length at fast speed. Horizontal lines represent the mean difference and the upper and lower limit of agreements (dotted line).

Table 1 contains means and standard deviations of FTSST duration, stride length and time as obtained from the three systems and reference tools; RMSEs are also given. High correlation, close to linearity (slopes differed from unity by on average 4 (SD 9) %), was found between parameters from the three systems and the gold standards (Figs. 2–5). Correlation was reduced at slow speeds (*r*^2^ < 0.8). Bland Altman plots for each parameter indicated a high level of agreement between the sensor and reference parameters. Mean difference and 95% confidence interval values are reported in the graphs (Figs. 2–5). Almost perfect agreement [25] was found between sensors for all parameters (ICC > 0.95, Table 1).

## 4 Discussion

The effectiveness of exercise therapy in managing knee OA is hampered by a lack of individualised management approaches and low adherence to exercise regimes. Introducing quantitative information on patients’ functional activity level and performance has the potential to enhance treatment compliance and inform personalised treatment. Three sensors were used in this study to monitor activities usually prescribed to OA patients. All three sensors demonstrated the capability to monitor the activities conducted with high comparability to the reference tools. The majority of data points were within the locus of agreement and showed a small bias. Moreover, the outcome measures were similar to those reported in the literature [11,15–20,26–28]. In line with previous studies, higher errors were noticed at lower speed [27,29]. The FTSST showed a higher RMSE and bias than walking tasks especially for the waist IMU. This could be attributed to the movement of the waistband as well as RPSIS marker, which was not directly attached on the skin and hence more prone to movement artefacts. Excellent agreement was also found when comparing sensors between each other as observed by high ICCs and thus demonstrating how these sensors offer the possibility to monitor simple markers of functional performance with clinical relevance in an accurate, easy, fast and unrestrictive manner without the need for expensive, bulky and time consuming laboratory equipment.

Sit-to-stand is a highly demanding activity often compromised in patients with knee OA. The FTSST is frequently used as a performance outcome measure to assess lower extremity strength and dysfunction [28,30,31]; the possibility to monitor patients performing such test remotely over long periods of time would allow tracking progression and adjusting intervention accordingly. Furthermore, such tools would facilitate motivating patients through personal targets to keep exercising thereby enhancing treatment compliance. The same applies to gait parameters often used for the assessment of patients’ disabilities [12,28]. We have demonstrated the validity of the wearable systems in providing quantitative measurements of participant functional status and current literature support the use of such measures to inform treatment interventions, either conservative or surgical, as well as to evaluate treatment outcomes for the long-term management of OA [28,31–35]. However, a limitation of the study is that we tested the systems with able-bodied participants only; further tests will be conducted to verify if the systems maintain their validity when used with knee OA population.

Accelerometers are frequently used to monitor activity, but we have demonstrated that a novel flexible sensor has the same potential. The flexible sensor system has been previously used to determine knee angles in an artificial setting during a quasi-static task [14]. Our results have demonstrated the capacity of the sensor to monitor knee function during dynamic tasks part of activities of daily living. As such, these strengths combined demonstrate the potential of this small and unobtrusive sensor to provide clinical and biomechanical relevant information of knee joint status that could be introduced to facilitate rehabilitation practice and patient monitoring. To determine joint angles from accelerometer data, multiple sensors are used which can be impractical for deployment with patients. Participants were asked their impressions on the systems used after completion of the test, and they reported how they liked the idea of having the technology integrated into their clothing and the feeling that “it was barely there”.

In addition, our results also suggest that a functional placement is a valid position for performance monitoring. The thigh IMU during the test was positioned to replicate a pocket placement but attached with tape. Users could place the system in their trousers pocket with minimum visibility and intrusiveness issues. Participants raised concerns that the waist IMU would be noticeable to others if worn for a prolonged period of time, whereas this was avoided if the system was hidden in the pocket. However, participants were also concerned about the bulkiness of the thigh IMU and complained that it obstructed arm swinging during walking. The thigh IMU was a first prototype of a wireless sensing node; design improvements are underway to reduce the dimensions of the system to increase acceptance.

The advantage of using a system in a functional position to identify physical performance means that there is potential to utilise the accelerometer embedded in most smartphones for monitoring physical function. This would allow the use of a device that is already highly integrated into most people's daily routine but developing further its clinical use (e.g., providing feedback on a clinically prescribed exercise routine).

Finally, we proposed the use of a method based on FFT to evaluate outcome measures with good success. Only one study was found to use a similar approach [18]. This method removes the necessity of identifying specific patterns and thresholds in accelerations trajectories that may be too specific for the overall population and impairments and, may be affected by misalignment and inaccurate positioning. The use of FFT approach was necessary, particularly for the novel sensor, as no clear pattern could be distinguished and related to particular movements for all the participants. The different fitting of the garment on each participant's knee may be related to that. On the other hand, this highlighted how slightly altering the sensor position will not compromise its outputs thus making it an easy system to wear.

## 5 Conclusion

OA is a widespread problem disabling our adult population. Measures need to be taken to change the paradigm by which exercises are administered to enhance their effectiveness. The use of wearable sensors provides the possibility to monitor patients while exercising over extended periods of time. Three sensors, two based on accelerometry with different placements and a novel sensor, based on conductive flexible material, were shown to be capable of monitoring activity performance. Although the clinical population of interest was knee OA population and only two activities were monitored, these systems could be used with other impaired groups and more clinical tests could be monitored using the same approach. Tests are now being conducted with the novel sensor in real life settings and focus groups and interviews are being conducted with OA patients and clinicians to explore their views and preferences on the use of wearable technology to maximise future clinical acceptance and guide the design of the novel system.

## Ethical approval

This study was approved by the Imperial College London Research Ethics Committee (ICREC_12_6_9).

## Conflict of interest

Nothing to declare.

## Figures and Tables

**Table 1:** Mean (SD), and RMSEs for FTSST and walking tasks evaluated parameters for the three systems (system 1: AWS; system 2: thigh IMU; system 3: waist IMU) used and reference tools. ICCs between sensors are also reported.

	System 1	System 2	Reference	System 3	Reference
FTSST task					
FTSST duration (s)	9.1 (2.0)	8.4 (1.9)	8.9 (2.0)	8.6 (1.9)	9.3 (1.9)
RMSE (s)	0.49	0.56		0.86	
ICC	0.9540				
Slow speed walking (0.61 (SD 0.04) m/s)					
Stride time (s)	1.50 (0.17)	1.51 (0.17)		1.50 (0.16)	1.56 (0.15)
RMSE (s)	0.09	0.09		0.09	
Stride length (m)	0.92 (0.13)	0.93 (0.13)		0.92 (0.13)	0.95 (0.13)
RMSE (m)	0.06	0.07		0.07	
Stride time ICC	0.9641			Stride length ICC	0.9755
Normal speed walking (1.23 (SD 0.08) m/s)					
Stride time (s)	1.10 (0.07)	1.09 (0.06)		1.09 (0.06)	1.11 (0.07)
RMSE (s)	0.02	0.02		0.02	
Stride length (m)	1.36 (0.14)	1.34 (0.13)		1.33 (0.12)	1.33 (0.14)
RMSE (m)	0.03	0.02		0.02	
Stride time ICC	0.9654			Stride length ICC	0.9881
Fast speed walking (2.05 (SD 0.35) m/s)					
Stride time (s)	0.88 (0.06)	0.87 (0.07)		0.86 (0.07)	0.88 (0.07)
RMSE	0.02	0.01		0.02	
Stride length (m)	1.78 (0.16)	1.78 (0.16)		1.77 (0.17)	1.77 (0.15)
RMSE (m)	0.03	0.03		0.02	
Stride time ICC	0.9586			Stride length ICC	0.9880

FTSST: five time sit-to-stand test; SD: standard deviation RMSE: root mean square error; ICC: intra class correlation coefficient.
